# OP11 How Has Structured Expert Elicitation Been Used To Provide Evidence For Holistic Value Elements In Health Technology Assessments?

**DOI:** 10.1017/S0266462325100706

**Published:** 2025-12-29

**Authors:** Ally Robert, Smarth Lakhanpal

## Abstract

**Introduction:**

Structured expert elicitation (SEE) is a method of formally collecting expert opinions and beliefs using a statistical framework that can be used to generate qualitative data for health technology assessment (HTA). SEE is a particularly useful strategy for rare diseases and innovative technologies, where clinical trial data can be subject to additional uncertainty.

**Methods:**

To investigate how SEE evidence has been used in HTA submissions for rare diseases, a targeted literature search was conducted to identify relevant submissions to the National Institute for Health and Care Excellence (NICE) in the UK via the highly specialized technology (HST) pathway. HST reports were screened to ensure that the company submission documents were publicly available and that details of any SEE studies were included. Fields of extraction included the methods used by SEE, the value drivers that SEE data justified, and the consideration of such evidence by the NICE decision-making committee in the final appraisal document (FAD).

**Results:**

All 27 submissions included in the final analysis leveraged SEE; eight submissions used two SEE methods, and 11 submissions used three methods. The most popular method was semi-structured interviews (81%), followed by surveys (59%), advisory boards (33%), and Delphi panels (26%). The majority of SEE evidence supported core value elements such as quality-adjusted life years (81%). However, SEE evidence was also used to support innovative value elements including family spillover (59%) and productivity (29%). Expert evidence was assessed in the majority of FADs but was accepted mostly when supporting core value elements.

**Conclusions:**

Commonalities in submissions that successfully leveraged SEE to support holistic value elements included providing clear justification for why the data could not be sourced elsewhere, conducting multiple formats of expert engagement, recruiting large samples of experts, and including a detailed overview of the SEE study design. Manufacturers should consider these critical success factors when including SEE in evidence generation strategies.

